# *Klebsiella pneumoniae:* a multidrug-resistant pathogen, has emerged in Saudi Arabia

**DOI:** 10.3389/fmicb.2025.1689974

**Published:** 2025-10-10

**Authors:** Jehad A. Aldali

**Affiliations:** Department of Pathology, College of Medicine, Imam Mohammad Ibn Saud Islamic University (IMSIU), Riyadh, Saudi Arabia

**Keywords:** mechanisms of resistance, multidrug-resistant, healthcare-associated infections, risk factors, Saudi Arabia

## Abstract

*Klebsiella pneumoniae* (*K. pneumoniae*), a significant opportunistic pathogen, has developed resistance mechanisms to numerous antimicrobials, including carbapenems. This article evaluates the prevalence, risk factors, antimicrobial susceptibility and resistance mechanisms of *K. pneumoniae* across various locations in Saudi Arabia. Hospital-acquired infections attributed to *K. pneumoniae* are prevalent in the country due to several factors, including the high incidence of critically ill patients, frequent gastrointestinal colonization and the extensive use of antimicrobial agents. The prevalence of *K. pneumoniae* strains resistant to multiple antimicrobials, including carbapenems, has risen. Hospitals facilitate the proliferation of multidrug-resistant (MDR) *K. pneumoniae* due to the extensive utilization of broad-spectrum antibiotics, the likelihood of interpatient transmission, the elevated risk of infection during invasive procedures in intensive care units and the frequent occurrence of invasive diagnostic and therapeutic interventions among diabetic and cancer patients. Combinations of colistin and tigecycline with carbapenems or other antibiotics remain the optimal treatment for patients with MDR *K. pneumoniae* infections, despite the increasing prevalence of resistance to these agents noted in numerous hospitals. The high incidence of MDR *K. pneumoniae* in Saudi hospitals necessitates comprehensive investigation into the molecular mechanisms underlying multidrug resistance. A thorough understanding of *K. pneumoniae* resistance patterns and the formulation of a treatment protocol to mitigate the infection burden in Saudi Arabia could be enhanced by establishing a local antibiogram database.

## Introduction

*Klebsiella pneumoniae (K. pneumoniae)* is a gram-negative, rod-shaped, non-motile bacterium from the *Enterobacteriaceae* family. This encapsulated and lactose-fermenting bacterium is part of the normal intestinal flora (Mohd Asri et al., 2021). It has a deoxyribonucleic acid (DNA) G+C composition of 56.7%–62.5%. A major virulence factor of *K. pneumoniae* is its polysaccharide capsule, which protects it from phagocytosis and desiccation. The capsule also facilitates biofilm formation, contributing to the bacterium’s persistence in both environmental and host settings (Ouellette et al., 1969), *K. pneumoniae* is commonly found in diverse environments such as soil, water, plants, and the gastrointestinal tracts of humans and animals. It is a significant pathogen, particularly in healthcare settings, where it can cause a range of infections, including pneumonia (especially in hospitalized patients on ventilators), wound infections characterized by redness, swelling and pus formation, soft tissue infections, and urinary tract infections. The mortality rate among patients with bloodstream infections (BSIs) caused by these organisms is notably high (Munoz-Price et al., 2013).

Several sophisticated molecular diagnostic procedures have been developed to accurately identify *K. pneumoniae*, including the analysis of the amplified 16S ribosomal ribonucleic acid (rRNA) gene’s restriction (Arenas et al., 2009). Other methods include transcription ribonucleic acid (tRNA) spacer fingerprinting, 16S-23S rRNA intergenic spacer restriction analysis, 16S-23S rRNA gene spacer sequence analysis (Wang et al., 2008), and rpoB (RNA polymerase β-subunit) gene sequencing and flanking spacers (Adékambi et al., 2009). Recent studies highlight the increasing prevalence and resistance patterns of *K. pneumoniae*, necessitating urgent public health interventions.

### Epidemiology

*K. pneumoniae* presents an infection control challenge for both nosocomial and community-acquired infections, accounting for one-third of gram-negative infections worldwide (Ah et al., 2014), The most common routes of transmission of *Klebsiella* spp. are through the gastrointestinal tract, medical equipment and the hands of medical personnel working in hospitals, due to their capacity to disseminate rapidly (Ahmed, 2021). Infected individuals may experience pneumonia, urinary tract infections and infections at surgical sites, making it one of the most common pathogens isolated in ICUs (Sokhn et al., 2020). The increasing prevalence of antibiotic-resistant strains has rendered *K. pneumoniae* more difficult to treat (Berglund, 2019). Consequently, this microbe is now acknowledged as a potentially catastrophic nosocomial infection that could result in a pandemic (Moriel et al., 2024). Moreover, multidrug-resistant (MDR) pathogens such as *K. pneumoniae* pose significant concerns for healthcare systems worldwide by complicating treatment and increasing morbidity, mortality, hospital stays and healthcare costs (Bassetti et al., 2018).

### Gene

Recent years have seen the emergence and spread of dominant ST2096 strains in the CC14 clade across hospital wards. On epidemiological timescales, these strains have developed resistance mutations against colistin, extended-spectrum beta-lactamase (ESBL) and carbapenemase genes (bla OXA-48 and bla OXA-232) on three plasmids. The mosaic plasmid containing the ESBL gene indicates that ST2096 strains are highly virulent. Colonization by ST2096 has been associated with sepsis and higher in-hospital mortality (Hala et al., 2024).

One study aimed to determine the global prevalence of the colistin resistance gene mutation. Several chromosome-linked gene mutations have been observed in the *mgrB, pmrB, phoQ, phoP*, and *pmrA* genes. Additionally, *mcr-1* and *mcr-8* genes have been acquired, as noted in certain eligible studies (Yusof et al., 2022). Colistin resistance in *K. pneumoniae* has been attributed to genetic mutation and plasmid acquisition. Regular assessment of mutation prevalence is crucial to prevent the spread of ColRkp and mitigate future risk due to its potential impact and lack of treatment options. Furthermore, commensal bacteria in food animals contain ColRkp of sequence types associated with human disease (ST11, ST37 and ST15), posing a significant risk as the pathogen can spread to humans through food chains or direct exposure (Di Tella et al., 2019).

Given the lack of effective antimicrobial therapy for ColRkp, it is essential to carefully review the chosen treatment. With few new antimicrobials available for the treatment of resistant healthcare-associated infections, effective prevention programs and adequate personnel are necessary to combat ColRkp. Rapid, low-cost and accurate detection of resistance factors and mutations is critical for routine molecular analyses and improved patient management. To enhance surveillance and management of infectious diseases, it is recommended to avoid unnecessary procedures and inappropriate use of antibiotics in healthcare settings. High rates of antimicrobial resistance pose a major global health risk. This study details the global prevalence of colistin resistance in *K. pneumoniae* isolates, including drug resistance genes, which will assist authorities in implementing measures to combat antimicrobial resistance (Yusof et al., 2022).

The prevalence of MDR *K. pneumoniae* has been increasing globally. In Europe, a study reported that 31.2% of *K. pneumoniae* isolates were resistant to at least one antibiotic class, with a significant rise in carbapenem resistance (Mohd Asri et al., 2021). In the United States, the Centers for Disease Control and Prevention (CDC) reported that carbapenem-resistant *Enterobacteriaceae* (CRE), including *K. pneumoniae*, is a major public health threat, with an estimated 13,100 infections and 1,100 deaths annually (Jimenez, 2020).

In Saudi Arabia, the situation mirrors global trends but with some regional specifics. Studies have shown a high prevalence of MDR *K. pneumoniae* in hospital settings.

### *K. pneumoniae* resistance mechanism

Most *K. pneumoniae* strains are now entirely resistant to broad-spectrum antibiotics used clinically, exhibiting several resistance mechanisms targeting different antibiotic classes. The first mechanism involves the production of beta-lactamases, which hydrolyse beta-lactam antibiotics, rendering them ineffective. Two notable resistant strains of *K. pneumoniae* are ESBL and carbapenem-resistant *K. pneumoniae* (CRKP). ESBL-producing *K. pneumoniae* can hydrolyse a wide range of beta-lactams, including penicillin and cephalosporins, which complicates treatment options. Conversely, CRKP is resistant to carbapenems, often considered the last line of defense against MDR bacteria. ESBL prevalence rates were reported to be higher, similar or lower in certain studies conducted in Saudi Arabia. Al-Kharaj and Al-Khobar indicate lower prevalence rates of ESBL-producing *K. pneumoniae*, at 10.4% and 12.2% (1,21), respectively, while Abha reported a prevalence rate of 27.5% (8). A high prevalence rate of ESBL-producing *K. pneumoniae* (55%) was observed in Riyadh (3). The variation in the prevalence rate of ESBL in Saudi Arabia may be attributable to differences in the type and volume of antimicrobial agent consumption and the timing of isolate collection (Tawfik et al., 2011).

Other mechanisms involve utilizing efflux pumps to eliminate antibiotics from the cell and modifying antibiotic target sites through genetic mutations. Deoxycholate, sodium dodecyl sulfate, and disinfectants such as benzalkonium chloride, chlorhexidine and triclosan demonstrate the transporter’s broad substrate specificity in *K. pneumoniae* (Srinivasan et al., 2014).

A major virulence factor of *K. pneumoniae* is the production of capsules and lipopolysaccharides. The complex biochemistry of these capsules protects the bacterium from phagocytosis and desiccation, which is vital for its pathogenicity, enhancing its survival in hostile environments and contributing to its ability to cause infections. Currently, *Klebsiella* strains produce over 77 distinct capsule antigens, which are used to differentiate strains during clinical infections (Clegg and Murphy, 2017).

Additionally, the genetic mechanisms of capsule production and synthesis of *K. pneumoniae* are noteworthy. The cps gene cluster, located near the gene on the bacterial chromosome, contains capsule-producing genes. The strain’s serotype is determined by this 15-kb DNA cluster, which encompasses all the genetic determinants for capsule biosynthesis. Important regulatory proteins such as RcsB and RmpA regulate cps gene expression. RcsB enhances gene transcription, while RmpA and its alleles regulate capsule production. An RmpA-induced hypermucous phenotype indicates increased capsule production. Environmental factors, particularly iron availability, also affect capsule production. In iron-rich conditions, the Fur protein inhibits RmpA and other virulence genes, reducing capsular polysaccharide production. This regulation illustrates how *K. pneumoniae* adapts to its environment.

Another important virulence factor is urease, which is an extracellular enzyme responsible for hydrolysing urea into ammonia and carbamate, and can adversely affect the urinary tract environment. The nickel-dependent enzyme requires accessory proteins (UreD, UreE, UreF, and UreG) to assemble. Urease production by *K. pneumoniae* raises local pH by producing ammonia, which may lead to urinary tract infections. This pH increase can precipitate inorganic salts, encrusting urinary catheters and other surfaces. Such encrustations can reduce urine flow, bacterial clearance and biofilm formation, thus complicating treatment and increasing infection risk.

### Antimicrobial resistance emerging in Riyadh and Al-Qassim, Saudi Arabia

The emergence of nosocomial MDR *K. pneumoniae* in numerous hospitals in Saudi Arabia has become a substantial healthcare and economic concern.

### Riyadh region

Saudi Arabia is divided into five primary regions: Riyadh, Eastern, Northern, Southern, and Western. Several reports exist in Saudi Arabia on ESBL production by *K. pneumoniae*. To determine the prevalence of ESBL.

In 2007, 220 *K. pneumoniae* samples were isolated from two hospitals in Riyadh and screened for ESBL production using ESBL-E-strips and combined disk methods. PCR was employed to detect bla_*TEM*_, bla_*SHV*_, and bla_*CTX–M*_ genes. The findings indicated that 55% of *K. pneumoniae* isolates were ESBL-positive by phenotypic analysis. ESBL-producing *K. pneumoniae* had PCR positive for SHV, TEM and CTX-M b-lactamase genes, with prevalence rates of 97.3, 84.1 and 34.1%, respectively. The CTX-M family contains two enzyme groups: CTX-M-1 and CTX-M-9-like genes, with 60 and 40% prevalence, respectively. The resistance rates for cefotaxime, ceftazidime and amoxicillin/clavulanate were 97% (215/220), 95% (210/220) and 86% (190/220), respectively. Cefepime, a fourth-generation cephalosporin, exhibited moderate activity (47%), while 4.5% (*n* = 10/220) were resistant to cefoxitin. All ESBL-producing isolates were susceptible to imipenem. The resistance to cefoxitin in these isolates may be due to changes in ompK35 or ompK36, rather than AmpC enzymes, as the MIC of b-lactam/b-lactamase inhibitors is significantly reduced.

Additionally, the co-existence of enzymes like OXA may reduce b-lactam/b-lactamase inhibitor susceptibility. High resistance to gentamicin and amikacin was observed in ESBL-producing isolates (88.9% [200/220] and 773.0% [170/220], respectively). However, they exhibited an 11% lower resistance rate to ciproflaxin. According to PCR assays, the prevalence of SHV, TEM and CTX-M genes in these isolates was 97.3% (*n* = 214/220), 84.1% (*n* = 185/220) and 34.1% (*n* = 75/220), respectively. PCR experiments revealed that 45 (60%) of 75 CTX-M-producing isolates carried blaCTX-M-1-like genes, while 30 (40%) carried blaCTX-M-9-like genes (Al-Agamy et al., 2009).

In a study conducted at King Abdulaziz Medical City (KAMC) in Riyadh, 1,706 *K. pneumoniae* isolates were detected between September 2009 and August 2010. A prolonged hospital stay (91%), indwelling devices (81%), surgical procedures (74%), carbapenem use (62%) and colonization/infection with other MDR organisms (MDROs) (57%) were experienced by most patients. Notably, 38% of patients with CRKP died during the current hospitalization, and 25% of them experienced clinical infection. To mitigate a CRSKP outbreak in the acute care setting, staff education, environmental cleaning, hand hygiene and contact isolation were implemented; however, these measures did not prevent endemicity (Balkhy et al., 2012).

A retrospective analysis, conducted at King Fahad Medical City in Riyadh, Saudi Arabia, with 152 *K. pneumoniae* isolates from January 2019 to January 2020, found that pediatric patients (33.6%) had a lower risk of infection than adult patients (66.4%). Additionally, the infection rate was slightly higher in women than in men, and neurological disorders were the primary cause of *K. pneumoniae* BSI across all ages. Most deaths occurred among adults with multi-organ dysfunction. *K. pneumoniae* also exhibited significant resistance to amoxicillin-clavulanate (72.4%), ceftazidime (67.8%), cephalothin (76.3%) and carbapenems (36.1%) (Hafiz et al., 2023).

Another study in Riyadh, conducted by the King Khalid University Hospital microbiology laboratory, collected samples from 2006 to 2010. Out of 17,105 samples, 1,076 (6.3%) were *Escherichia coli* (808) and *K. pneumoniae* (268) ESBL-producing isolates. In urine samples, 680 (63.2%) isolates were found, followed by 287 (26.7%) in superficial, deep wounds, tissues and sterile body fluids, 71 (6.6%) in respiratory samples, and 38 (3.5%) in blood. The frequency of ESBL *E. coli* was 6.6%, while *K. pneumoniae* accounted for 5.5%. Over the study period, ESBL-producing *E. coli* and *K. pneumoniae* increased significantly. Resistance rates for ciprofloxacin (68.2%), gentamicin (47%) and cotrimoxazole (71.1%) were observed in *E. coli*. Similarly, 62.7% of *K. pneumoniae* isolates were gentamicin-resistant, 59.5% were cotrimoxazole-resistant, and 49.8% were ciprofloxacin-resistant. Antimicrobial resistance did not change significantly during the study (Somily et al., 2014).

In Riyadh, a study conducted from June to December 2011 found *K. pneumoniae* isolates with reduced sensitivity to carbapenems. Only non-duplicate clinical and surveillance isolates from inpatients were included. Extended-length PCR was performed to detect spectrum beta-lactamase genes (blaCTX-M, blaTEM, blaSHV) and carbapenemase genes (blaKPC, blaVIM, blaIMP, blaNDM, blaOXA-48). Susceptibility to imipenem, meropenem, amikacin, gentamicin, trimethoprim-sulfamethoxazole and colistin was assessed. Of the 60 *K. pneumoniae* isolates studied, 45 were from ICU patients. Moreover, 47 isolates had blaOXA-48, 12 had blaNDM, and one had blaVIM. Neither did an isolate have a combination of these resistance genes, nor did they have blaKPC or blaIMP. Each of the 37 blaCTX-M-positive isolates belonged to CTX-M group 1, while 29 were positive for both blaCTX-M and blaOXA-48. A total of 17 and 39 isolates had blaTEM and blaSHV genes, respectively, indicating a high level of resistance to trimethoprim-sulfamethoxazole, ciprofloxacin, gentamicin and amikacin. Three isolates were identified as having resistance to colistin and were positive for blaOXA-4 (Shibl et al., 2013).

A 16-month investigation from July 2011 to October 2012, collected 4,250 gram-negative, non-duplicate bacterial isolates from clinical samples of patients at El Iman Hospital in Riyadh. Of these, 3,358 (79%) were *E. coli* and 892 (21%) were *K. pneumoniae*. Strains were collected from various body fluids, including blood, urine, wounds and sputum. Most samples were obtained from urine (69%) or blood (21%). The isolates frequently exhibited two or three types of broad-spectrum b-lactamases.

The study found blaVIM (297/4250, 7%) and blaIMP (382/4250, 9%) in 55% (2338/4250) of isolates, along with the blaNDM gene. KPC and OXA-48 were not detected in our isolates. The majority of ESBL-producing isolates were of the CTX-M type. At least one bla gene was found in 66.6% (2830/4250) of isolates. The blaCTX-M, blaTEM and blaSHV genes were found in 63.5% (2698/4250), 58% (2465/4250) and 27.3% (1169/4250) of the isolates, respectively. The isolates exhibited a combination of blaCTX-M and blaTEM in 55% (2335/4250) and blaCTX-M and blaTEM-blaSHV in 18.8% (805/4250). Among the CTX-M types, CTX-M 9 (57%) and CTX-M 1 (32%) were more common than CTX-M 2 (11%). A high rate of co-resistance to other antibiotics was observed in all isolates, which were also resistant to imipenem and meropenem. Three isolates were identified as blaOXA-48-positive and resistant to colistin (Marie et al., 2013).

### Al-Qassim region

#### Antibiotic resistance patterns

The study reported concerning levels of antibiotic resistance among the *K. pneumoniae* isolates (190 isolates). The resistance rates were as follows: 64.75% for cefazolin, 62.63% for trimethoprim/sulfamethoxazole, 59.45% for ampicillin, 58.42% for cefoxitin, 57.37% for ceftriaxone, 53.68% for cefepime, 52.11% for ampicillin-sulbactam, 50.53% for ceftazidime, 52.11% for ertapenem and 49.47% for imipenem. These findings highlight the significant threat posed by MDR *K. pneumoniae*. The double-disk synergy test indicated that 48.95% of *K. pneumoniae* isolates were classified as ESBL producers (Marzouk et al., 2024).

In another study conducted between January and June 2008, 430 clinical isolates of *K. pneumoniae* were collected from general ward inpatients in two hospitals in Buraydah, Al-Qassim. ESBL prevalence was 25.6% (110/430), with all isolates sensitive to imipenem and tigecycline, but resistance to gentamicin, amikacin and ciprofloxacin was 87.3, 10 and 9.1%, respectively. Among these strains, 89.1, 70.9 and 36.4% produced SHV, TEM and CTX-M, respectively. The prevalence of ESBL SHV-12 and SHV-5 was 60 and 18.2%, respectively. Non-ESBL SHV also occurred, including SHV-1 (5.5%), SHV-11 (3.6%) and SHV-85 (1.8%). However, CTX-M-15 prevalence was 34.5%, and CTX-M-14 prevalence was 1.8%. ISEcp1 was found in 60% of blaCTX-M-15 genes. Although all blaCTX-M genes were transferable, most blaSHV-12 and blaSHV-5 were not. TEM-type ESBLs were not found in any isolates. This study represents the first Saudi Arabian description of CTX-M-14, SHV-5, SHV-11, and SHV-85. It was found that *K. pneumoniae* SHV-12 dominates, while CTX-M-15 is emerging in Saudi Arabia (Tawfik et al., 2011). Table 1 summarizes the data from studies conducted in the Riyadh and Al-Qassim regions.

**TABLE 1:** Studies in Riyadh and Al-Qassim report high resistance rates in *K. pneumoniae* isolates.

Study	City	Period	Number of samples	Prevalence of MDR *K. pneumoniae* /percentage	Genes	References
Two hospitals	Riyadh	During 2007	220	Cefotaxime, ceftazidime, and amoxicillin/clavulanate were 97%. Gentamicin and amikacin resistance was found in ESBL-producing isolates (88.9% [200/220] and 773.0% [170/220]).	SHV, TEM, and CTX-M	Al-Agamy et al., 2009
King Abdulaziz Medical City	Riyadh	Between September 2009 and August 2010	1706	38% of patients with carbapenem-resistant *K. pneumoniae* (CRKP) died during the current hospitalization	N/A	Balkhy et al., 2012
King Fahad Medical City	–	January 2019 to January 2020	152	Resistance to amoxicillin-clavulanate (72.4%), ceftazidime (67.8%), cephalothin (76.3%), and carbapenems (36.1%)	N/A	Hafiz et al., 2023
King Khalid University Hospital	Riyadh	2006 to 2010	17105	Gentamicin-resistant, 59.5% cotrimoxazole-resistant, and 49.8% ciprofloxacin	N/A	Somily et al., 2014
Hospital in Riyadh	Riyadh	June to December 2011	60	A high level of resistance to trimethoprim–sulfamethoxazole, ciprofloxacin, gentamicin, and amikacin. Three isolates were identified as having resistance to colistin and were positive for blaOXA-4.	Harbored blaOXA-48, 12 were positive for blaNDM, and one for blaVIM	Shibl et al., 2013
El-Iman Hospital	Riyadh	July 2011 to October 2012	892	Imipenem- and meropenem- resistant. blaOXA-48-positive isolates with colistin resistance were detected.	Combination of blaCTX-M and blaOXA-48. blaTEM and blaSHV genes blaOXA-48	Marie et al., 2013
various hospitals in the Al-Qassim	Al-Qassim	March 2021 and October 2022	190	The resistance rates: 64.75% for cefazolin, 62.63% for trimethoprim/sulfamethoxazole, 59.45% for ampicillin, 58.42% for cefoxitin, 57.37% for ceftriaxone, 53.68% for cefepime, 52.11% for ampicillin-sulbactam, 50.53% for ceftazidime, 52.11% for ertapenem, and 49.47% for imipenem.	N/A	Marzouk et al., 2024
Two hospitals in Buraydah	Buraydah	January–June 2008	430	Sensitive to imipenem and tigecycline, but resistance to gentamicin 87.3%, amikacin 10%, and ciprofloxacin was 9.1%	N/A	Tawfik et al., 2011

#### Antimicrobial resistance emerging in the Southern Region of Saudi Arabia includes Aseer and Jazan Region

Aseer is the southern Saudi Arabian capital. A study evaluated the extent of K. pneumoniae infections and explored the corresponding antimicrobial resistance profile over 10 years (from January 2018 to December 2019). The study examined the prevalence, susceptibility to antimicrobials and impact of *K. pneumoniae* bacteria on clinical outcomes at Aseer Central Hospital. *K. pneumoniae* (*n* = 276) caused 39% of ICU infections, followed by *Acinetobacter* spp. (30%), *Pseudomonas aeruginosa* (10.0%), *E. coli* (7%), and others (14%). A 33.3% mortality rate was observed among the 276 ICU patients, with 42% of cases linked to *K. pneumoniae*, and 67% of deaths occurring in individuals aged 50–90.

*K. pneumoniae* was highly sensitive and recommended for in vivo treatment with tigecycline (81%), cefazolin (77.2%), colistin (64.9%) and, to a lesser extent, norfloxacin (60%) and imipenem (55.5%). High resistance was found for ampicillin (100%), ESBL-SCM (100%), piperacillin (100%) and ceftazidime (92.5%). Higher carbapenem resistance was observed in ertapenem (65.2%) and meropenem (61.7%). The rise in *K. pneumoniae* infections poses a threat to ICU patients, having been linked to nearly half of ICU deaths. Using tigecycline alone or in combination with colistin at high doses may be more effective in treating CRKP infections (Al Bshabshe et al., 2020).

In another study, a retrospective analysis was conducted at a tertiary hospital in Aseer. This hospital serves as a reference and educational institution, receiving patients from the Aseer region – both directly and through referrals. All complete and eligible records from 2013 to 2022 were reviewed. A total of 3,921 isolated *K. pneumoniae* samples were collected from 28,420 bacterial samples. The isolation rate started at 11.3% in 2013, dropped to 6.1% in 2016, peaked at 16.3% in 2021, and then decreased to 12.8% in 2022. In total, 23.7% of *K. pneumoniae* samples were found in urine, 19% in sputum, 14% in wounds and 11.7% in blood samples. The antibiotic resistance rate of *K. pneumoniae* increased significantly from 2013 to 2022, especially in 2020 and 2021, before decreasing again in 2022. The resistance rate decreased from 22.2% in 2013 to 18.6% in 2016, then increased to 54.6 and 56.4% in 2020 and 2021, respectively (*p* = 0.039) (Alshehri et al., 2024).

Another cross-sectional study took place from late April to September 2015 in Aseer. Fifty-four *K. pneumoniae* isolates with reduced carbapenem sensitivity were obtained from clinical specimens at the two largest hospitals in the Southern province. The MICs of carbapenems were confirmed through an *E*-test. The most common carbapenemase genes (blaIMP, bla-carbapenem-hydrolyzing oxacillinase [OXA-48], blaVIM, bla-New Delhi metallo-ß-lactamas [NDM] and blaKPC) were detected using multiplex PCR. The results indicated that CRKP isolation increases with age and ICU admission. Most carbapenemases (81.5%, *n* = 44) were OXA-48, suggesting an endemic prevalence. In 7.4% (*n* = 4) of isolates, NDM was the second most common carbapenemase, while only one isolate had Verona integron-encoded VIM (Al-Zahrani and Alsiri, 2018).

A separate study conducted in the southern region of Saudi Arabia, specifically in Jazan, collected 86 non-repetitive clinical isolates of *K. pneumoniae* that were carbapenem-resistant. This was done from March 2020 to April 2021 across three Jazan tertiary hospitals. Several automated systems identified and tested isolates for antimicrobial susceptibility (AST). Using multiplex PCR, carbapenemase genes were detected. Of the 86 CRKP isolates tested, 64 (74.4%) produced carbapenemase. The most common carbapenemase gene was blaOXA-48, found in 65.1% (*n* = 56) of isolates. Only 9.3% (*n* = 8) of isolates had blaNDM, and three had blaVIM. Interestingly, one CRKP isolate had carbapenemase genes (blaNDM, blaVIM and blaKPC) associated with COVID-19 (Brek et al., 2023). Table 2 summarizes the data from studies conducted in the Aseer and Jazan regions.

**TABLE 2:** Studies in South of Saudi Arabia report high resistance rates in *K. pneumoniae* isolates.

Study	City	Period	Number of samples	Prevalence of MDR *K. pneumoniae* /percentage	Genes	References
Aseer Central Hospital	Aseer	2013–2022	276	High-sensitivity and recommended for in vivo treatment of tigecycline (81%), cefazolin (77.2%), colistin (64.9%), norfloxacin (60%) and imipenem (55.5%). Ampicillin (100%), ESBL-SCM (100%), piperacillin (100%), and ceftazidime (92.5%) were resistant. Ertapenem (65.2%) and meropenem (61.7%) had higher carbapenem resistance. *K. pneumoniae* infections, which cause nearly half of ICU deaths, are rising. Tigecycline alone or in combination with colistin at high doses may treat carbapenem-resistant *K. pneumoniae* infections better.	N/A	Al Bshabshe et al., 2020
Tertiary hospital in Aseer	Aseer	2013–2022	28,420	*K. pneumoniae* antibiotic resistance increased from 2013 to 2022, especially in 2020 and 2021, before decreasing in 2022. The resistance rate dropped from 22.2% in 2013 to 18.6% in 2016, then rose to 54.6% and 56.4% in 2020 and 2021.	N/A	Alshehri et al., 2024
Two largest hospitals in the Southern province.	Aseer	Late April to September 2015	54	The majority of carbapenemases (81.5%, *n* = 44) were OXA-48, suggesting an endemic prevalence. New Delhi metallo-ß-lactamas (NDM) was the second most common carbapenemase in 7.4% (*n* = 4) of isolates, while only one isolate tested positive for Verona integron-encoded VIM.	blaIMP blaVIM, bla-New Delhi metallo-ß-lactamas [NDM], ablaKPC, blaOXA48	Al-Zahrani and Alsiri, 2018
Three Jazan tertiary hospitals	Jazan	March 2020 to April 2021	86	Dissemination of carbapenem resistance	blaOXA-48, blaNDM, blaVIM. (blaNDM, blaVIM, and blaKPC	Brek et al., 2023

Antimicrobial resistance emerging in the western region of Saudi Arabia includes Medina, Makkah, Jeddah and Taif.

#### Medina region

A study examined the prevalence and trends of the antibiotic resistance of *K. pneumoniae* at King Fahad Hospital in Medina from February 27, 2014, to December 31, 2018. A total of 15,708 isolates was taken from 1,149 patients. An unprecedented rise in carbapenem resistance was observed, with 38.4% (*n* = 436) for imipenem and 46.1% (*n* = 371) for meropenem, the first treatment recommended by local guidelines. Additionally, colistin and tigecycline, commonly used alternatives, also exhibited high resistance rates (40.7% (*n* = 105) for colistin and 53.3% (*n* = 220) for tigecycline). Furthermore, third and fourth-generation cephalosporins showed resistance rates ranging from 57.5 to 77.8%. Co-resistance with imipenem was over 75% for other management options, except for colistin and tigecycline, which had rates of 53.6% (*n* = 89) and 61.4% (*n* = 167), respectively. In conclusion, the study found increased resistance to beta-lactams, carbapenems, and alternative treatments such as colistin and tigecycline (Al-Zalabani et al., 2020).

#### Makkah region

In the western region, Makkah is the most important city, attracting millions of Muslims from around the world for Umrah and pilgrimage. A retrospective study was conducted at a tertiary hospital in Makkah, collecting data from January 2011 to December 2021. A total of 61,027 bacterial isolates were collected from clinical samples, with 14.7% (*n* = 9,014) being *K. pneumoniae*. During the study period, *K. pneumoniae* exhibited a significant increase in antibiotic resistance across most tested antibiotics.

Resistance rates for amoxicillin/clavulanate and piperacillin/tazobactam increased significantly from 33.6 and 13.6% in 2011 to 71.4 and 84.9% in 2021. Ceftazidime, cefotaxime and cefepime resistance rates rose from 29.9, 26.2 and 53.9% in 2011 to 84.9, 85.1 and 85.8% in 2021, respectively. Additionally, imipenem and amikacin resistance rates increased significantly, from 6.6% in 2011 to 59.9 and 62.2% in 2021, respectively. The study also found that *K. pneumoniae* prevalence and drug resistance increased over time and concluded that to control and reduce antimicrobial resistance, hospital-acquired infections had to be prevented and antibiotics were to be used responsibly (Jalal et al., 2023).

In another study conducted in the Makkah region between January 2017 to December 2017 across four main tertiary care hospitals, out of 120 confirmed *Enterobacteriaceae* isolates, *K. pneumoniae* and *E. coli* were the most prevalent, accounting for 35 and 34.2% of the infections, respectively. Carbapenem resistance was observed in 26 isolates (21.7%), with *K. pneumoniae* making up the majority (21 out of 26). Notably, 17 of these isolates carried triple resistance genes (KPC/NDM-1/OXA-48), while the remaining four isolates carried dual resistance genes (KPC/OXA-48 or NDM-1/OXA-48). The study found a significantly higher incidence of these triple resistance genes among males (COR 4.5; CI: 1.9–17.3; *P* = 0.018), non-Saudis (COR 4.9; CI: 1.5–19.3; *P* = 0.003), in specimens obtained from ICUs (COR 3.6; CI: 1.5–8.4; *P* = 0.002) and in blood samples (COR 2.8; CI: 1.1–6.9; *P* = 0.02). MDR *Enterobacteriaceae*, particularly *K. pneumoniae* harboring KPC, NDM-1 and OXA-48 genes, are emerging in the Western region of Saudi Arabia. This study marks the first recorded instance of *K. pneumoniae* co-producing triple carbapenemase genes associated with enterobacterial infections in this region (Khan et al., 2019).

Another prospective study was conducted in five hospitals in Makkah from January to July 2015, where 260 clinical isolates of *K. pneumoniae* were obtained. VITEK 2 identified all clinical isolates as *K. pneumoniae*. These isolates were then screened for carbapenemase producers by determining reduced susceptibility to carbapenems using representative antibiotic disks of third-generation cephalosporins and carbapenems in the disk diffusion test according to Clinical and Laboratory Standards Institute guidelines. The minimum inhibitory concentration of screening-positive isolates for cephalosporins, carbapenems, aminoglycosides, tigecycline and colistin was determined using VITEK 2. A modified Hodge test was used to detect carbapenemase production in suspected isolates. Out of 260 *K. pneumoniae* isolates, 31 (11.9%) were 100% cephalosporin-resistant. Carbapenemase was found in 15 (48.4%) of the 31 *K. pneumoniae* isolates. All carbapenem-resistant isolates were sensitive to colistin and tigecycline; however, these isolates exhibited resistance to amikacin (41.9%) and gentamicin (51.6%) (Khan and Faiz, 2016).

At a Saudi Arabian tertiary hospital in Jeddah, a study conducted from 2014 to 2018 screened 286 MDR *Klebsiella* spp. isolates for resistance patterns. AST testing was conducted on all isolates using VITEK 2 and broth microdilution. Resistance-conferring genes were identified using Illumina shotgun sequencing and PacBio SMRT sequencing protocols. The novel ST-3510 strain of *K. quasipneumoniae* subsp. *similipneumoniae*, a CRE, carries a blaKPC-2 carbapenemase-encoding gene. The isolate NGS-421 was obtained from surveillance of 286 clinical isolates of MDR *Klebsiella* spp. using shotgun Whole Genome Sequencing (WGS). In late 2017, the NGKPC-421 isolate was recovered from a septic patient and initially misidentified as *K. pneumoniae*. After sequencing and assembling the NGKPC-421 genome, a 39.4 kb IncX6 plasmid with a blaKPC-2 gene and transposable elements (ISKpn6-blaKPC-2–ISKpn27) was identified (Hala et al., 2019).

Another study conducted at the King Abdulaziz Specialist Hospital in Taif, Saudi Arabia, recovered 30 *K. pneumoniae* isolates from patients. Blood, sputum, urine and wound swabs yielded strains. The study tested 22 antibiotics against *K. pneumoniae* isolates, revealing high variability in resistance. All isolates were resistant to ampicillin (100%) and highly resistant to piperacillin (93.33%), while the lowest resistance was observed for imipenem (13.33%). Among male patient isolates, ampicillin (100%) and piperacillin (85.71%) had the highest resistance, with weaker resistance to fosfomycin and tigecycline (21.42%). Female isolates showed resistance to all antibiotics except imipenem, with the highest resistance to ampicillin and piperacillin (100%). No significant gender-based differences in resistance or ESBL production were found. Specimen-based analysis showed urine and blood isolates were resistant to all antibiotics except imipenem, with 100% resistance to ampicillin and piperacillin. Sputum isolates were resistant to all antibiotics except fosfomycin, with the highest resistance to ampicillin (100%). Wound swab isolates were resistant to all antibiotics except tigecycline. MDR was found in 83.33% of isolates, with some strains resistant to up to 20 antibiotics. PCR analysis identified the acrAB efflux pump gene in 93.33% of isolates, mdtK in 10%, tolC in 83.33%, and ompK35 and ompK36 in 76.66 and 90%, respectively. Molecular typing using (GTG)5 and BOX-PCR showed clustering patterns, with (GTG)5 significantly correlating with antibiotic resistance patterns (Lagha et al., 2021). Table 3 summarizes data from studies conducted in the Medina, Makkah, Jeddah and Taif regions.

**TABLE 3:** Studies in western region of Saudi Arabia report high resistance rates in *K. pneumoniae* isolates.

Study	City	Period	Number of samples	Prevalence of MDR *K. pneumoniae* /percentage	Genes	References
King Fahad Hospital in Medina	Medina	February 27, 2014, to December 31, 2018	15708	Carbapenem resistance 4%, imipenem and 46.1%, meropenem, the first treatment recommended by local guidelines. Additionally, colistin and tigecycline, commonly used alternatives, also had high resistance colistin and 53.3%.	N/A	Al-Zalabani et al., 2020
Makkah tertiary hospital	Makkah	January 2011 to December 2021	9014	Resistance rates for amoxicillin/clavulanate and piperacillin/tazobactam increased significantly. Ceftazidime, cefotaxime, and cefepime resistance rates. imipenem and amikacin resistance rates increased significantly.	N/A	Jalal et al., 2023
Four tertiary care hospitals	Makkah	January 2017 to December 2017	120	The prevalence of carbapenem resistance was observed in various Enterobacteriaceae species. Thus, *K. pneumonia* was the most prevalent strain (80.8%).	KPC, NDM-1 and OXA-48	Khan et al., 2019
Five Makkah hospitals	Makkah	January to July 2015	260	100% cephalosporin-resistant 31 (11.9%). 15 (48.4%) of 31 *K. pneumoniae* isolates had carbapenemase. All carbapenem-resistant isolates were colistin/tigecycline-sensitive. These isolates are amikacin (41.9%) and gentamicin (51.6%) resistant.	N/A	Khan and Faiz, 2016
Tertiary hospital	Jeddah	2014–2018	286	The study emphasizes the significance of using rapid sequencing technologies in clinical microbiology for accurate taxonomic classification and antimicrobial resistance monitoring.	ST-3510 blaKPC-2 NGKPC-421	Hala et al., 2019
Abdulaziz Specialist Hospital	Taif	N/A	30	All isolated was resistant to ampicillin (100%) and piperacillin (93.33%), but imipenem was the least resistant (13.33%). Male patient isolates were most resistant to ampicillin (100%) and piperacillin (85.71%), with fosfomycin and tigecycline (21.42%) being less resistant. Girls were resistant to all antibiotics except imipenem.	acrAB mdtK, olC, ompK35, ompK36,	Lagha et al., 2021

#### Bisha region

A prospective cross-sectional study was conducted in King Abdullah Hospital (KAH) located in Bisha over a period from April 2021 to March 2022. *K. pneumoniae* (*n* = 211) bacteria were tested for antibiotic susceptibility in clinical samples from adult patients. Univariate and multivariate logistic regressions were used to identify factors linked to MDR *K. pneumoniae* infection. MDR *K. pneumoniae* strains were found in 66.8% (142/211) of patients and exhibited the highest resistance rates for ampicillin (100%), cefuroxime (97.9%), ceftriaxone (94.3%) and aztreonam (92.2%). The lowest resistance rates were found for colistin (16.3%) and tigecycline (6.4%). Patient factors such as gender, age, ICU admission, invasive medical devices and chronic illness were significantly linked to MDR *K. pneumoniae* infection. Male gender, age ≥65, ICU admission, diabetes and chronic obstructive pulmonary disease were independent risk factors for MDR *K. pneumoniae* infection (AOR 2.107, 95% CI 1.125–3.945, *p* = 0.02). The study provided insight into MDR *K. pneumoniae* infection in our setting and suggested future research to prevent and reduce MDR bacteria (Ibrahim, 2023).

A study conducted in the western region of Saudi Arabia – Al-Hofuf – analyzed 52 *K. pneumoniae* isolates. Several of these were classified as extensively drug-resistant (XDR), including AB70, AB76, AB97 and AB93, which exhibited 100% resistance to multiple antimicrobial agents. Among these, only AB97 was from an ICU patient. Other XDR isolates included AB59, AB64, AB67, AB69, AB95, AB96 and K107, with K107 being collected 2 years earlier than the others. Most remaining isolates (65%) were MDR or had moderate susceptibility (4%). All isolates were resistant to amoxicillin, with high resistance observed for ampicillin/sulbactam (96.4%), amoxicillin/clavulanic acid (91%), cefoxitin (82.6%), ceftazidime (83.3%) and aztreonam (80%). Carbapenems (ertapenem, meropenem and imipenem) showed better effectiveness, though resistance varied, with 23 and 28% resistance to imipenem and meropenem, respectively, and only 5.5% to ertapenem. Notably, 98% of isolates were ESBL producers, and one (K1) was resistant to colistin. Resistance genes were widespread, with ISAba1 being the least detected insertion sequence. OXA-23 was found in 67% of isolates, while metallo-β-lactamases (SIM and IMP) were present in 72.5%. Among ESBL genes, SHV and CTX co-existed in 43% of isolates, while TEM was the least detected (Badger-Emeka et al., 2021).

## Conclusion

In conclusion, the spread of *K. pneumoniae* in Saudi Arabia is significantly influenced by the complex nature of the hospital setting, patient comorbidities, length of hospital stay, ICU complexity, concurrent diseases and antimicrobial agent use. Numerous Saudi Arabian tertiary referral hospitals have identified that *K. pneumoniae* has emerged as increasingly resistant to a variety of antimicrobial drugs. Municipal health departments may encounter this challenge, as it has been reported in numerous regions. Nevertheless, there is a dearth of information in certain smaller communities, as well as in the North. Consequently, extensive monitoring systems, the assistance of a national antimicrobial stewardship program and an infection prevention initiative are required in Saudi Arabia to expand the understanding of *K. pneumoniae* resistance patterns.
